# Barriers and Facilitators to eHealth Use in Daily Practice: Perspectives of Patients and Professionals in Dermatology

**DOI:** 10.2196/jmir.7512

**Published:** 2017-09-05

**Authors:** Lieneke FM Ariens, Florine ML Schussler-Raymakers, Cynthia Frima, Annebeth Flinterman, Eefje Hamminga, Bernd WM Arents, Carla AFM Bruijnzeel-Koomen, Marjolein S de Bruin-Weller, Harmieke van Os-Medendorp

**Affiliations:** ^1^ Department of Dermatology and Allergology University Medical Center Utrecht Utrecht Netherlands; ^2^ Diakonessenhuis Utrecht Netherlands; ^3^ Dutch Association for People with Atopic Dermatitis Nijkerk Netherlands

**Keywords:** dermatology, eHealth, implementation, barriers, facilitators

## Abstract

**Background:**

The number of eHealth interventions in the management of chronic diseases such as atopic dermatitis (AD) is growing. Despite promising results, the implementation and use of these interventions is limited.

**Objectives:**

This study aimed to assess opinions of the most important stakeholders influencing the implementation and use of eHealth services in daily dermatology practice.

**Methods:**

The perspectives of health care professionals and patients towards the implementation and use of eHealth services in daily practice were assessed by using a mixed method design. A cross-sectional survey based on the eHealth implementation toolkit (eHit) was conducted to explore factors influencing the adoption of eHealth interventions offering the possibility of e-consultations, Web-based monitoring, and Web-based self-management training among dermatologists and dermatology nurses. The perspectives of patients with atopic dermatitis (AD) regarding the use of eHealth services were discussed in an online focus group.

**Results:**

Health care professionals (n=99) and patients (n=9) acknowledged the value of eHealth services and were willing to use these digital tools in daily dermatology practice. Key identified barriers (statements with <50% of the participants scoring totally agree or agree) in the implementation and adoption of eHealth interventions included concerns about the availability (12/99, 12%) and allocation (14/99, 14%) of resources, financial aspects (26/99, 26%), reliability, security, and confidentially of the intervention itself (29/99, 29%), and the lack of education and training (6/99, 6%).

**Conclusions:**

Health care professionals and patients acknowledge the benefits arising from the implementation and use of eHealth services in daily dermatology practice. However, some important barriers were identified that might be useful in addressing the implementation strategy in order to enhance the implementation success of eHealth interventions in dermatology.

## Introduction

In recent years, the increasing prevalence and care of chronic diseases has become a growing burden on modern health care systems. To maintain availability and enhance efficiency of health care, there has been an increasing focus on the development and value of eHealth interventions in the management of chronic diseases [1-5].

Within dermatology, different eHealth interventions are available for use in daily practice. Teledermatology is the most well-known eHealth intervention and has been widely implemented in daily dermatology care [6,7]. The number of health care services combining Web-based accessibility of medical records, systems for interaction between health care professionals and patients (e-consultations), and patient education in the management of chronic skin diseases is growing [8-14]. However, given the promising results on cost-effectiveness, such interventions are not as widely used as might be expected. To implement innovations in daily health care practice, a phase-based approach tailored to specific groups and settings is most successful [15]. The first step of such an approach includes a context analysis to explore factors influencing the implementation. Based on previous research [16-25], different factors affecting the implementation and adoption of eHealth interventions from the perspective of patients and health care providers were identified, including the technological context, product features, and the user and organizational context.

The eHealth implementation toolkit (eHit) is a tool with a phased approach, which can be used to implement eHealth innovations [26]. The design of this tool is based on available evidence about eHealth implementation including data from systematic reviews of barriers and facilitators to implementation of eHealth initiatives, qualitative data derived from interviews of implementers, and the normalization process theory (NPT). The NPT [27] is a conceptual framework for implementing and embedding complex interventions in everyday work. The eHit is described as a sensitizing tool, presenting factors that need consideration before an eHealth intervention can be integrated into daily practice. Assessing attitudes towards the implementation of eHealth interventions and acceptance by relevant stakeholders could provide important information and might enhance the implementation success.

This study aimed to assess the attitudes of relevant stakeholders towards the implementation and adoption of eHealth interventions in daily dermatology practice. A cross-sectional survey based on the eHit [26] was conducted to explore factors affecting the adoption of interventions offering the possibility of e-consultations, Web-based monitoring, and Web-based self-management training among dermatologists and nurses. Patients’ participation in their health care is recognized as a key component in the quality of health care. Besides, as an end user of eHealth interventions, patient’s involvement at different levels in the implementation process gives valuable insights and may improve the implementation and use of eHealth interventions in daily practice [28,29]. Therefore, perspectives of patients with atopic dermatitis (AD) concerning the use of eHealth interventions were discussed in an online focus group.

## Methods

### Design

To address perspectives of different stakeholders, a concurrent triangulation mixed method design was used. A cross-sectional survey based on the eHit was performed among Dutch dermatologists and nurses to explore their opinions concerning barriers and facilitators for implementation and adoption of eHealth interventions. In the Netherlands, the following eHealth applications are available for use in daily practice: (1) patient portals offering the possibility of interaction between health care professionals and patients (e-consultations); (2) Web-based monitoring of the disease by using digital photographs and insight in the medical record; and (3) self-management trainings focusing on treatment adherence, prevention of exacerbations, and coping with itch and psychological problems. Furthermore, perspectives of adult AD patients and parents of children with AD regarding the use of such eHealth interventions in daily dermatology practice were discussed in a qualitative study in an online focus group.

### Sampling and Recruitment

Dutch dermatologists were purposively recruited to participate in the survey anonymously, via an email invitation by the authors sent by the Dutch Association for Dermatologists (NVDV) or personal email invitation. Members of a platform for nurses, which is aimed to increase the expertise of nursing care for patients with AD or itch, were approached to be included in this study. Additionally, dermatologists and nurses were recruited from an academic and regional hospital in the Netherlands, participating in an implementation project for digital care in patients with AD.

The qualitative part of this study contained an online focus group including adult AD patients and parents of young children with AD (aged 0-8 years). Participants were recruited by 2 research nurses at the outpatient clinic of the dermatology department of the University Medical Center Utrecht (UMCU) and through an advertisement by the Dutch Association for People with Atopic Dermatitis (VMCE). Purposive sampling was performed to include participants with and without experience with digital tools.

### Outcome Parameters

#### EHit Survey

A survey based on the eHit [26] was developed to explore barriers and facilitators for implementation and adoption of an eHealth intervention among dermatologists and nurses. This Web-based questionnaire contained 23 statements with a 5-point Likert scale ranging from 1 (completely disagree) to 5 (completely agree), which were grouped into 3 main sections.

1. Context: national and hospital policy, attitudes of professionals, resources, and risks
2. Intervention: consequences for professional – patient relationship, safety, ease of use, and benefits and cost–effectiveness
3. Workforce: consequences for work load, collaboration, work patterns, and training and responsibility.

The survey used in this study based on the eHit was not validated.

### Topics and Procedure in the Online Focus Group

Perspectives of AD patients and parents of children with AD were explored by using an online focus group. In 2 weeks, 8 statements were posted in the online focus group on Facebook to explore patients’ opinions concerning experiences with digital tools in health care, the usefulness of digital contact with other patients and e-learning, advantages and disadvantages of digital tools, and the willingness and requirements for the use of such digital tools in daily practice.

The online focus group was set up in a closed account on Facebook for a 2-week period. After the informed consent was obtained, participants were invited to join the online focus group by the researcher or research nurse. The discussion in the online focus group was started by the researcher giving an explanation considering the aim of the discussion and posting the first statement. Participants were motivated to react on the statements and to participate actively in the group discussion. The researcher and research nurse asked for clarifications and experiences, asked questions, and made summaries. The researcher and research nurse were both involved in the development and research of eHealth interventions. The research nurse was also involved in the patient care of two participants however the researcher was not connected with the participants. The quantitative and qualitative part of this study did not fall under the scope of the Medical Research Involving Human Subjects, which was confirmed by the Medical Research Ethics Committee of the UMC Utrecht, the Netherlands for the qualitative part including patients.

### Analysis

Results of the eHit survey were analyzed using IBM SPSS Statistics Version 21 (SPSS Inc, Chicago, IL, USA). Answers on the 5-point Likert scale ranging from 1 (completely disagree) to 5 (completely agree) were categorized into 3 categories: totally disagree or disagree, totally agree or agree, and unknown. The mean number of participants who totally agreed or agreed was calculated per statement of each category (context, intervention, and workforce). Statements with <50% of the participants scoring totally agree or agree were considered to be a barrier for implementation and adoption of an eHealth intervention. Statements with >50% of the participants scoring totally agree or agree were considered to be a facilitator. Results are shown for the total group of professionals and categorized in professionals with and without eHealth experience. Differences in responses between health care professionals with and without eHealth experience, medical doctors, and nurses were analyzed by using the Pearson's chi-squared test.

A generic qualitative analysis was used to analyze the discussion yield in the online focus group including coding, categorizing, formulizing themes, and connecting and interpreting them [30]. To ensure trustworthiness, interpretations and conclusions were summarized before closing the online focus group to conduct a member check. The researcher and research nurse both coded all text and discussed the analyses and results until consensus was achieved.

## Results

### EHit Survey

In total 800 health care professionals were approached to complete the survey, of which 108 responded, yielding a response rate of 14%. The survey response rates among members of de NVDV, the nurses’ platform, and the participating hospitals were 48/670 (7%), 24/69 (35%), and 36/61 (59%), respectively. Reasons for not responding on the questionnaire are unknown. Respondents with another profession than dermatologist or nurse were excluded from the analysis. As shown in Table 1, 99 health care professionals who completed the eHit survey were included of which 65 (66%) were dermatologists and 34 nurses (34%). Out of the 99 participants, 65 (66%) were female and the mean age (SD) of the total group of professionals was 47 (SD 10.5). A total of 26 dermatologists and 16 nurses reported no experience in digital care compared to 39 dermatologists and 18 nurses with experience in digital care. The 23 statements used in the survey and the respondents scores on agreement are fully presented in Multimedia Appendix 1.

**Figure 1:**
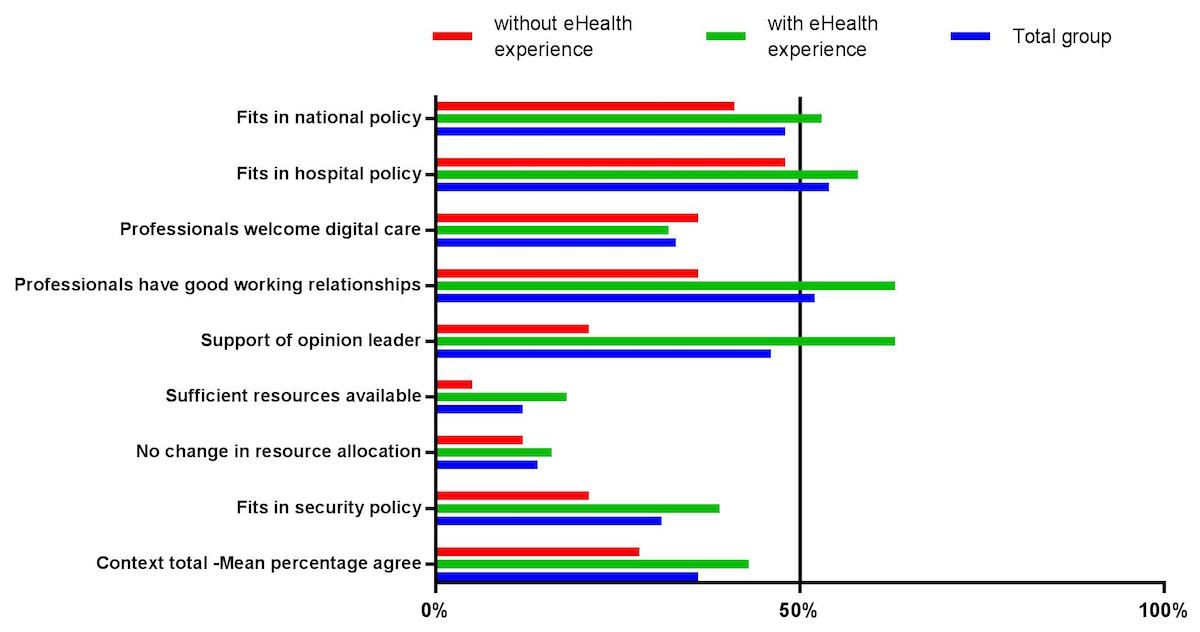
Percentage of participants scoring totally agree or agree on statements related to the context.

The mean number of participants that totally agreed with statements related to the context was 36% (Figure 1). Context related barriers (<50% of the respondents scoring totally agree or agree) included the availability of resources (12 /99, 12%), their allocation (14/99, 14%), security policy (31/99, 31%), and the organizational culture welcoming eHealth initiatives (33/99, 33%). Context related facilitators (≥50% of the respondents scoring totally agree or agree) included “fits in local policy regarding efficiency and patient-centered care” (53/99, 54%) and “good working relationships between different professionals involved in the implementation of digital care” (51/99, 52%). Among the professionals with eHealth experience, the highest scoring facilitator was “presence of particular opinion leaders who are likely to support the implementation of digital care” (36/57, 63%).

**Figure 2:**
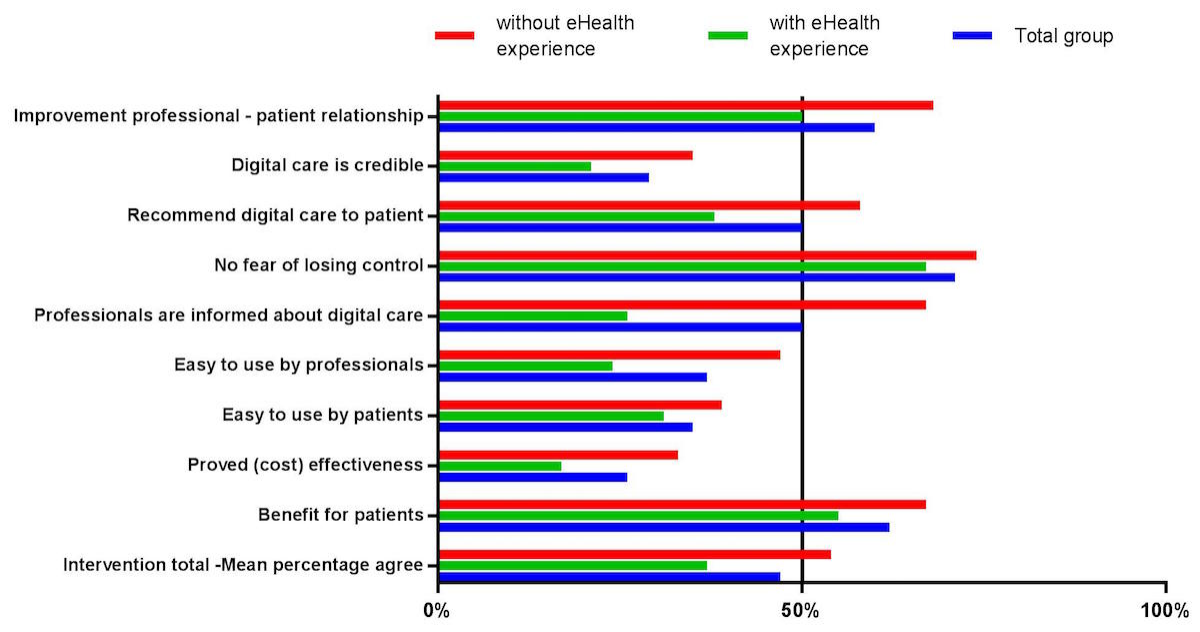
Percentage of participants scoring totally agree or agree on statements related to the intervention.

A total of 47% of respondents totally agreed with statements related to the intervention (Figure 2). Credibility of digital care in terms of confidentiality, security, and reliability of the intervention (29/99, 29%), ease of use for patients (35/99, 35%) and professionals (37/99, 37%), and the cost-effectiveness of eHealth interventions (26/99, 26%) were identified as barriers for implementation. Facilitators included “facilitation of health care professional – patient interaction” (59/99, 60%), benefits for patients (61/99, 62%), and the notion of professionals that they do not fear losing control when using e-consultations instead of face-to-face visits (70/99, 71%).

**Table 1:** Characteristics of respondents.

Characteristics		Total (n=99)	No experience with digital care (n=42)		Experience with digital care (n=57)	
			Dermatologist (n=26)	Nurse (n=16)	Dermatologist (n=39)	Nurse (n=18)
**Gender, n (%)**						
	Male	34 (34)	12 (46)	0 (0)	22 (56)	0 (0)
	Female	65 (66)	14 (54)	16 (100)	17 (44)	18 (100)
Age (missing n=1), mean (SD)		47.0 (10.5)	45.0 (12.6)	50.4 (6.6)	46.1 (10.9)	48.7 (8.8)
**Organization, n (%)**											
	General hospital	50 (51)	12 (46)	12 (75)	16 (41)	10 (56)
	University hospital	37 (37)	13 (50)	2 (13)	16 (41)	6 (33)
	Primary care or home health care or other	5 (5)	0 (0)	2 (13)	1 (3)	2 (11)
	Independent treatment centre	7 (7)	1 (4)	0 (0)	6 (15)	0 (0)
**Experience with digital care, n (%)**						
	E-consultation	23 (23)	0 (0)	0 (0)	15 (39)	8 (44)
	Patient portal	16 (16)	0 (0)	0 (0)	7 (18)	9 (50)
	Web-based self-management training	14 (14)	0 (0)	0 (0)	4 (10)	10 (56)
	Teledermatology	18 (18)	0 (0)	0 (0)	18 (46)	0 (0)
	Video of webcam consult	2 (2)	0 (0)	0 (0)	1 (3)	1 (6)
	Other^a^	9 (9)	0 (0)	0 (0)	8 (21)	1 (6)

^a^Other eHealth tools such as wound-telemonitoring system, portal for Web-based questionnaires, informational site.

**Figure 3:**
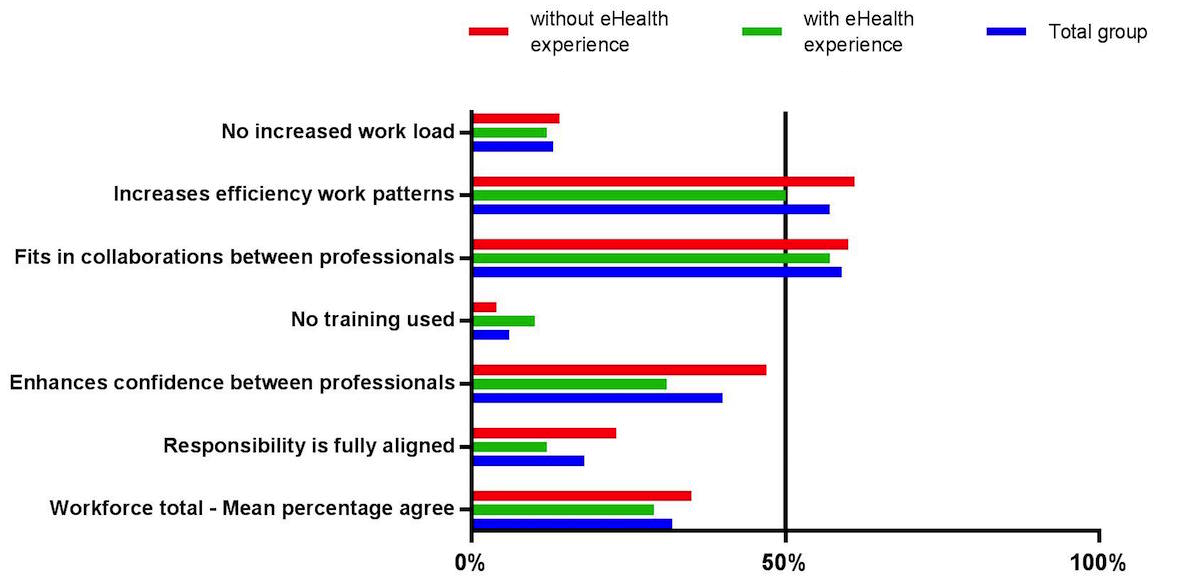
Percentage of participants scoring totally agree or agree on statements related to the workforce.

Finally, the percentage of participants totally agreeing with statements related to workforce comprised 32% (Figure 3). Barriers for implementation related to workforce included concerns about increased workload (13/99, 13%), the need for training prior to the implementation of digital care (6/99, 6%), and the alignment of responsibility for the use of digital care (18/99, 18%). The possible increase of efficiency in work patterns (56/99, 57%) and collaboration between health care professionals (58/99, 59%) were identified as facilitators.

As shown in Multimedia Appendix 1, the respondents’ scores on agreement significantly differed between professionals with and without experience in using eHealth interventions for the statements “nurses and medical doctors involved in the implementation of digital care have good working relationships, good communication and co-operation” (*P*=.01), and “there are particular opinion leaders within the hospital who are likely to support the implementation of digital care” (*P*<.001) related to the context. Agreement scores related to the intervention were significantly different among experienced and non-experienced professionals for the statements “I will recommend my patients to use digital care” (*P*=.004), “I know what digital care comprises and how it can be used” (*P*<.001), “digital care is easy to use by medical doctors and nurses” (*P*=.001) and “digital care has been well evaluated and has been demonstrated to improve health care in a cost effective manner” (*P*=.02). Considering workforce, the agreement of health care professionals on the statement “workload during (future) implementation is not increased” (*P*=.008) significantly differed between experienced and nonexperienced professionals. Professionals with experience in digital care were more likely to totally agree with the above-mentioned statements compared to professionals without experience in digital care.

### Online Focus Group

A total of 15 AD patients or parents of children with AD were approached to participate in the online focus group. No response to the invitation, lack of experience with Facebook, and privacy concerns were the main reasons not to participate in the online focus group. In total, 7 patients with AD and 2 parents of children with AD were included (Table 2). Out of the 9 participants, 8 were female and 1 was male.

### Digital Tools in Health Care: Advantages and Disadvantages

Most patients were not experienced in using digital tools. However, discussing the value of e-consultation as a potential digital tool, all patients acknowledged its value and were willing to use these tools in daily practice. Reported advantages included the possibility of sending photographs and request e-repeat prescriptions, the quick and satisfying response to questions, and the fact that it is available on any weekday without making an appointment. The possibility of contacting a health care professional by using e-consulting at the time of exacerbations is the most important advantage reported by the participants. By using e-consultations, patients expect to receive effective care at the moment it is most useful and needed. Sending photographs makes it possible for health care professionals to access disease activity and give treatment advice. Furthermore, an e-consultation can eliminate an extra face-to-face contact. It is timesaving and patient friendly:

...e-consultations are a valuable addition. It is not to cut costs but to increase the quality of treatment.

ID04

Insight in the medical record as one of the functionalities in the electronic patients’ portal was appreciated by patients who used this digital tool. Patients acknowledged the possibility to review the altered treatment again.

The advantage is that you can read the report again if needed.

ID06

The possibility to gain insight in their medical record and read the status reported by the caregiver made them feel more confident with their disease and treatment.

Participants also reported disadvantages of the Web-based tools. Patients think that there will be less personal contact with the caregiver:

A disadvantage of using an e- consultation is that you will increasingly depend on a computer to control your disease via the Internet. The personal (social) contact with your caregiver decreases.

ID07

Besides, patients are not willing to pay extra costs for this digital tool and have concerns about the privacy and safety of digital care. They see the possibility of using an e-consultation as complementary, not as a replacement of face-to-face consultations: “I think a healthy alternation between face-to-face consultations and e- consultations is necessary.” (ID09)

### Other Digital Tools: Digital Contact With Other Patients and E-Learning

Participants acknowledged the value of having digital contact with other patients by using a digital forum or chat area. They mentioned the usefulness of sharing experiences with fellow patients, especially in combination with the medical point of view from the caregiver. Patients endorsed the value of the Web-based e-learning program substance but noticed there is already a lot of information available on the Internet. Websites referred by the caregiver were considered to be more reliable:

If a caregiver refers to a website, it’s more reliable in my eyes.

ID05

**Table 2:** Characteristics of participants of the online focus group.

Characteristics	ID	Sex	Age	Educational level^a^	Internet use	Internet skills
**University hospital - Children’s department**						
	1	F	37	Medium	(almost) daily	Good
	2	F	33	High	(almost) daily	Good
**University hospital - Adult’s department**						
	3	F	19	Medium	(almost) daily	Good
	4	F	24	High	(almost) daily	Moderate
	5	F	55	Low	Several times a week	Moderate
**Dutch Association for People with Atopic Dermatitis (VMCE)**						
	6	F	34	High	(almost) daily	Very good
	7	F	45	Low	(almost) daily	Good
	8	F	21	Medium	(almost) daily	Good
	9	M	59	High	(almost) daily	Very good

^a^Educational level: low, elementary education, high school or middle-level applied education; high: higher professional or academic education.

## Discussion

The results of this study illustrate the overall positive attitude of health care professionals and patients towards the implementation and use of eHealth services offering the possibility of e-consultations, Web-based monitoring, and Web-based self-management training in daily dermatology practice. However, some remaining challenges were also identified. Both health care professionals and patients acknowledge the value of eHealth services and are willing to use such interventions in daily practice. Patients appreciate the comprehensive accessibility of digital care and the possibility to gain insight in the medical record and to contact a health care professional in times of exacerbations. Besides, health care professionals value the potential increase of efficiency in work patterns and collaborations. Key identified barriers in the implementation and adoption of eHealth interventions included concerns about financial aspects, reliability, security, confidentiality, and cost-effectiveness of the intervention itself as well as the lack of education and training.

These barriers are in line with findings from current literature [18-21,23,31-33]. In different studies investigating the implementation and adoption of a variety of eHealth interventions in a range of settings, technological knowledge, and skills: financial aspects, social and organizational support, and the lack of education and training are the most frequently noted barriers [18-21,23,31-33]. Crucial factors leading to the successful implementation of teledermatology have been the focus on embedding the intervention in the existing health care infrastructure, the comprehensive support and training of future users, and the clear assignment of persons who took full responsibility for the entire process [34]. Besides, user satisfaction was identified as an important factor in the implementation process [34]. These findings demonstrate that, despite methodological differences, studies identified quite similar factors that should be considered before implementing eHealth interventions in daily practice.

An interesting finding of this study is the more positive attitude towards implementation and use of eHealth services among professionals with experience in using eHealth applications. Experienced health care professionals acknowledge the advantages arising from eHealth services and report fewer barriers in the implementation process as compared to those who are not experienced in using such services. Previous research investigating the opinions of health care professional towards the introduction of a new eHealth service in Sweden also showed a significantly more positive attitude among experienced professionals compared to inexperienced professionals [35]. These findings imply that professionals need to overcome some obstacles to enable them to recognize potential benefits that can be derived from implementation of eHealth interventions. Receiving education and training in the implementation process might help to lower the threshold and increase acceptance of eHealth interventions in daily dermatology practice [15].

Results of the online focus group demonstrate a positive attitude towards to adoption and use of eHealth interventions in daily dermatology practice among AD patients. Previous research demonstrates that the acceptance of eHealth interventions among patients suffering from chronic diseases relies on their attitude towards the usefulness of Internet in personal health care [24]. In this study, most patients were not experienced in using eHealth applications. Interestingly, a lack of experience seemed not to negatively influence the acceptance of eHealth interventions. Compared with other studies assessing the attitude of patients towards the introduction of eHealth services [21-25,33], we found a relatively positive attitude towards the use of digital use in daily practice. This might be explained by the recruitment of a relatively small group of patients in a tertiary hospital and through an advertisement by the Dutch Association for People with Atopic Dermatitis (VMCE). It is possible that patients who are interested in eHealth were more likely to respond, resulting in an overestimation of the positive attitude. Besides, one of the research nurses recruiting patients for the online focus group at the outpatient clinic of the dermatology department of UMCU was also involved in the patient care of 2 participants. Therefore, potential sample selection bias might have been introduced in this group. Additionally, patients recruited in a tertiary hospital are likely to have a more severe AD, which might have influenced their attitude towards the adoption and use of eHealth services.

In this study, the eHit was used to develop a survey to explore the attitude of health care professionals. The eHit is a tool with a phased approach that was designed based on present evidence about the implementation of eHealth interventions [26]. The use of the eHit was considered to be feasible and acceptable by a variety of professionals and use in different health care systems [36]. Therefore, the results of this survey combined with the results from the online focus group provide a good reflection of factors that need consideration before implementing and embedding interventions in daily practice.

A research limitation of the present study is the low response rate to the eHit survey, which may not fully represent the target population. Besides, the highest response rate was found among professionals recruited from hospitals participating in an implementation project for digital care in patients with AD. Possibly, health care professionals who are interested in eHealth or are already working with such interventions might be more likely to complete the survey, leading to response bias.

In conclusion, this study attempts to use a systematical method to provide attitudes and factors influencing the implementation and adoption of eHealth services in daily dermatology practice of key stakeholders. The overall attitude towards the adoption and use of eHealth services among health care professionals and AD patients was positive; however, we also identified some important challenges in the implementation process. Findings of this study might be useful in addressing the implementation strategy to the health care professionals’ and patients’ values, needs, and attitudes and consequently enhance the implementation success of eHealth interventions in daily dermatology.

## Appendix

Multimedia Appendix 1 Results of the eHit survey.

## Abbreviations

AD: atopic dermatitis
eHit: eHealth implementation toolkit
NVDV: Dutch Association for Dermatologists
UMCU: University Medical Center Utrecht
VMCE: Dutch Association for People with Atopic Dermatitis (VMCE)
