# Supplementary material for: Barriers and Facilitators to eHealth Use in Daily Practice: Perspectives of Patients and Professionals in Dermatology
Source: J Med Internet Res. 2017 Sep 5;19(9):e300. doi: 10.2196/jmir.7512 (PMC5605757; doi:10.2196/jmir.7512)
Supplement: Multimedia Appendix 1 [file jmir_v19i9e300_app1.pdf]

Supplementary table 1: Results of the eHit survey

Context-total		Mean per statement	Medical doctor	Nurse	p-value	Without experience	With experience	p-value
	(Totally) disagree	16 (18%)	12 (20%)	5 (15%)	0.799[1]	6 (16%)	10 (19%)	0.146[1]
	No agree/no disagree/ unknown	42 (46%)	26 (44%)	16 (50%)		22 (57%)	20 (38%)	
	(Totally) agree	33 (36%)	22 (37%)	11 (35%)		10 (27%)	23 (43%)	
Context -Policy		Total	Medical doctor	Nurse	p-value	Without experience	With experience	
The use of digital care is compatible with current and planned national policy	(Totally) disagree	13 (13%)	6 (9%)	7 (21%)	0.205[1]	3 (7%)	10 (18%)	0.053[1]
	No agree/no disagree/ unknown	39 (39%)	25 (39%)	14 (41%)		22 (52%)	17 (30%)	
	(Totally) agree	47 (48%)	34 (52%)	13 (38%)		17 (41%)	30 (53%)	
The use of digital care is compatible with the hospital policy regarding efficiency and patient-centred care	(Totally) disagree	9 (9%)	6 (9%)	3 (9%)	0.595[1]	4 (10%)	5 (9%)	0.583[1]
	No agree/no disagree/ unknown	37 (37%)	22 (34%)	15 (44%)		18 (43%)	19 (33%)	
	(Totally) agree	53 (54%)	37 (57%)	16 (47%)		20 (48%)	33 (58%)	
Context - Culture		Total	Medical doctor	Nurse	p-value	Without experience	With experience	
Nurses and medical doctors welcome digital care	(Totally) disagree	25 (25%)	20 (31%)	5 (15%)	0.189[1]	10 (24%)	15 (26%)	0.905[1]
	No agree/no disagree/ unknown	41 (41%)	26 (40%)	15 (44%)		17 (40%)	24 (42%)	
	(Totally) agree	33 (33%)	19 (29%)	14 (41%)		15 (36%)	18 (32%)	
Nurses and medical doctors involved in the implementation of digital care have good working relationships, good communication and co-operation	(Totally) disagree	10 (10%)	6 (9%)	4 (12%)	0.414[1]	4 (10%)	6 (11%)	**0.013**[1]
	No agree/no disagree/ unknown	38 (38%)	28 (43%)	10 (29%)		23 (55%)	15 (26%)	
	(Totally) agree	51 (52%)	31 (48%)	20 (59%)		15 (36%)	36 (63%)	
The use of digital care is supported by a project leader who will provide time	(Totally) disagree	2 (7%)	2 (11%)	0 (0%)	0.449[1]	1 (11%)	1 (5%)	0.427[1]

and energy to promote the implementation	No agree/no disagree/ unknown	15 (54%)	10 (56%)	5 (50%)		6 (67%)	9 (47%)	
	(Totally) agree	11 (39%)	6 (33%)	5 (50%)		2 (22%	9 (47%)	
There are particular opinion leaders within the hospital who are likely to support the implementation of digital care	(Totally) disagree	8 (8%)	6 (9%)	2 (6%)	0.388[1]	5 (12%)	3 (5%)	**0.000**[1]
	No agree/no disagree/ unknown	46 (47%)	27 (42%)	19 (56%)		28 (67%)	18 (32%)	
	(Totally) agree	45 (46%)	32 (49%)	13 (38%)		9 (21%)	36 (63%)	
Context -Resources		Total	Medical doctor	Nurse	p-value	Without experience	With experience	
The hospital is well resourced and can fully meet the costs and additional workload resulting from the implementation of digital care (training, on-going support, contingencies and publicity)	(Totally) disagree	31 (31%)	23 (35%)	8 (24%)	0.464[1]	12 (29%)	19 (33%)	0.093[1]
	No agree/no disagree/ unknown	56 (57%)	35 (54%)	21 (62%)		28 (67%)	28 (49%)	
	(Totally) agree	12 (12%)	7 (11%)	5 (15%)		2 (5%)	10 (18%)	
The use of digital care will make no change to existing allocation of resources (money, staff, time and room)	(Totally) disagree	37 (37%)	27 (42%)	10 (29%)	**0.045**[1]	14 (33%)	23 (40%)	0.557[1]
	No agree/no disagree/ unknown	48 (48%)	26 (40%)	22 (65%)		23 (55%)	25 (44%)	
	(Totally) agree	14 (14%)	12 (19%)	2 (6%)		5 (12%)	9 (16%)	
Context - Risks		Total	Medical doctor	Nurse	p-value	Without experience	With experience	
The use of digital care is compatible with the hospital's existing risk management policies	(Totally) disagree	11 (11%)	9 (14%)	2 (6%)	0.484[1]	1 (2%)	10 (18%)	**0.003**[1]
	No agree/no disagree/ unknown	57 (58%)	36 (55%)	21 (62%)		32 (76%)	25 (44%)	
	(Totally) agree	31 (31%)	20 (31%)	11 (32%)		9 (21%)	22 (39%)	
Intervention-total		Mean percentage per statement	Medical doctor	Nurse		Without experience	With experience	
	(Totally) disagree	16 (17%)	14 (22%)	2 (6%)	0.125[1]	5 (12%)	11 (19%	**0.041**[1]

	No agree/no disagree/ unknown	36 (37%)	22 (34%)	15 (44%)		21 (51%)	15 (27%)	
	(Totally) agree	46 (47%)	29 (44%)	17 (51%)		15 (37%)	31 (54%)	
Intervention – Impact on clinical practice		Total	Medical doctor	Nurse	p-value	Without experience	With experience	
The use of digital care will facilitate healthcare professional –patient interaction. It improves communication within consultations or enables faster completion of objectives	(Totally) disagree	14 (14%)	12 (19%)	2 (6%)	0.090[1]	6 (14%)	8 (14%)	0.165[1]
	No agree/no disagree/ unknown	26 (26%)	19 (29%)	7 (21%)		15 (36%)	11 (19%)	
	(Totally) agree	59 (60%)	34 (52%)	25 (74%)		21 (50%)	38 (67%)	
The use of digital care is credible in terms of confidentiality, security and reliability	(Totally) disagree	20 (20%)	18 (28%)	2 (6%)	**0.020**[1]	7 (17%)	13 (23%)	0.145[1]
	No agree/no disagree/ unknown	50 (51%)	32 (49%)	18 (53%)		26 (62%)	24 (42%)	
	(Totally) agree	29 (29%)	15 (23%)	14 (41%)		9 (21%)	20 (35%)	
I will recommend my patients to use digital care	(Totally) disagree	14 (14%)	14 (22%)	0 (0%)	**0.014**[1]	3 (7%)	11 (19%)	**0.004**[1]
	No agree/no disagree/ unknown	36 (36%)	21 (32%)	15 (44%)		23 (55%)	13 (23%)	
	(Totally) agree	49 (50%)	30 (46%)	19 (56%)		16 (38%)	33 (58%)	
I am not afraid to lose control and will not be uncertain when patients send an e-consult instead of visiting the outpatient clinic	(Totally) disagree	9 (9%)	9 (14%)	0 (0%)	0.073[1]	2 (5%)	7 (12%)	0.122[1]
	No agree/no disagree/ unknown	20 (20%)	12 (19%)	8 (24%)		12 (29%)	8 (14%)	
	(Totally) agree	70 (71%)	44 (68%)	26 (77%)		28 (67%)	42 (74%)	
Intervention – Ease of use		Total	Medical doctor	Nurse	p-value	Without experience	With experience	
I know what digital care comprises and how it can be used	(Totally) disagree	24 (24%)	19 (29%)	5 (15%)	0.247[1]	12 (29%)	12 (21%)	**0.000**[1]

	No agree/no disagree/ unknown	26 (26%)	15 (23%)	11 (32%)		19 (45%)	7 (12%)	
	(Totally) agree	49 (50%)	31 (48%)	18 (53%)		11 (26%)	38 (67%)	
Digital care is easy to use by medical doctors and nurses	(Totally) disagree	18 (18%)	16 (25%)	2 (6%)	0.071[1]	4 (10%)	14 (25%)	**0.001**[1]
	No agree/no disagree/ unknown	44 (44%)	27 (42%)	17 (50%)		28 (67%)	16 (28%)	
	(Totally) agree	37 (37%)	22 (34%)	15 (44%)		10 (24%)	27 (47%)	
Digital care is easy to use by patients	(Totally) disagree	20 (20%)	14 (22%)	6 (18%)	0.884[1]	7 (17%)	13 (23%)	0.390[1]
	No agree/no disagree/ unknown	44 (44%)	28 (43%)	16 (47%)		22 (52%)	22 (39%)	
	(Totally) agree	35 (35%)	23 (35%)	12 (35%)		13 (31%)	22 (39%)	
Intervention - Cost-effectiveness		Total	Medical doctor	Nurse	p-value	Without experience	With experience	
Digital care has been well evaluated and has been demonstrated to improve health care in a cost effective manner	(Totally) disagree	18 (18%)	17 (26%)	1 (3%)	**0.013**[1]	5 (12%)	13 (23%)	**0.024**[1]
	No agree/no disagree/ unknown	55 (56%)	31 (48%)	24 (71%)		30 (71%)	25 (44%)	
	(Totally) agree	26 (26%)	17 (26%)	9 (27%)		7 (17%)	19 (33%)	
Patients can benefit from digital care instead of usual care	(Totally) disagree	11 (11%)	10 (15%)	1 (3%)	**0.010**[1]	3 (7%)	8 (14%)	0.094[1]
	No agree/no disagree/ unknown	27 (27%)	12 (19%)	15 (44%)		16 (38%)	11 (19%)	
	(Totally) agree	61 (62%)	43 (66%)	18 (53%)		23 (55%)	38 (67%)	
Workforce-total		Total-mean percentage	Medical doctor	Nurse	p-value	Without experience	With experience	
	(Totally) disagree	32 (32%)	23 (35%)	9 (27%)	0.570[1]	11 (27%)	20 (35%)	0.285[1]
	No agree/no disagree/ unknown	36 (36%)	24 (36%)	12 (35%)		19 (44%)	17 (30%)	

	(Totally) agree	32 (32%)	19 (29%)	13 (38%)		12 (29%)	20 (35%)	
Workforce - Workload		Total	Medical doctor	Nurse	p-value	Without experience	With experience	
Workload during (future) implementation is not increased	(Totally) disagree	42 (42%)	31 (48%)	11 (32%)	0.301[1]	11 (26%)	31 (54%)	**0.008**[1]
	No agree/no disagree/ unknown	44 (44%)	27 (42%)	17 (50%)		26 (62%)	18 (32%)	
	(Totally) disagree	13 (13%)	7 (11%)	6 (18%)		5 (12%)	8 (14%)	
Digital care will increase the efficiency of current work patterns	(Totally) disagree	16 (16%)	12 (19%)	4 (12%)	0.474[1]	5 (12%)	11 (19%)	0.105[1]
	No agree/no disagree/ unknown	27 (27%)	19 (29%)	8 (24%)		16 (38%)	11 (19%)	
	(Totally) agree	56 (57%)	34 (52%)	22 (65%)		21 (50%)	35 (61%)	
Digital care is completely compatible with current division of labour and co-operation between nurses and medical doctors	(Totally) disagree	12 (12%)	9 (14%)	3 (9%)	0.758[1]	5 (12%)	7 (12%)	0.953[1]
	No agree/no disagree/ unknown	29 (29%)	19 (29%)	10 (29%)		13 (31%)	16 (28%)	
	(Totally) agree	58 (59%)	37 (57%)	21 (62%)		24 (57%)	34 (60%)	
Workforce - Education and training		Total	Medical doctor	Nurse	p-value	Without experience	With experience	
No training will be needed by any staff prior to the implementation of digital care	(Totally) disagree	73 (74%)	48 (74%)	25 (74%)	0.997[1]	30 (71%)	43 (75%)	0.462[1]
	No agree/no disagree/ unknown	20 (20%)	13 (20%)	7 (21%)		8 (19%)	12 (21%)	
	(Totally) agree	6 (6%)	4 (6%)	2 (6%)		4 (10%)	2 (4%)	
Workforce - Relationships		Total	Medical doctor	Nurse	p-value	Without experience	With experience	
Digital care will enhance confidence about each other's expertise and performance between different professional groups.	(Totally) disagree	16 (16%)	14 (22%)	2 (6%)	0.065[1]	7 (17%)	9 (16%)	0.227[1]
	No agree/no disagree/ unknown	43 (43%)	29 (45%)	14 (41%)		22 (52%)	21 (37%)	
	(Totally) agree	40 (40%)	22 (34%)	18 (53%)		13 (31%)	27 (47%)	
Responsibility for the use of digital care is fully aligned	(Totally) disagree	30 (30%)	21 (32%)	9 (27%)	0.300[1]	10 (24%)	20 (35%)	0.086[1]
	No agree/no disagree/ unknown	51 (52%)	35 (54%)	16 (47%)		27 (64%)	24 (42%)	

	(Totally) agree	18 (18%)	9 (14%)	9 (27%)		5 (12%)	13 (23%)	

[1] p-value obtained with the Pearson Chi-Square test
